# Catalytic Interface of rGO-VO_2_/W_5_O_14_ Hydrogel for High-Performance Electrochemical Water Oxidation

**DOI:** 10.3390/gels11080670

**Published:** 2025-08-21

**Authors:** Mrunal Bhosale, Rutuja U. Amate, Pritam J. Morankar, Chan-Wook Jeon

**Affiliations:** School of Chemical Engineering, Yeungnam University, 280 Daehak-ro, Gyeongsan 712-749, Republic of Korea

**Keywords:** electrochemical water splitting, oxygen evolution reaction, rGO-VO_2_/W_5_O_14_, hydrogel

## Abstract

The continuous increase in global energy demand necessitates the development of sustainable, clean, and highly efficient methods of energy generation. Electrochemical water splitting, comprising hydrogen evolution reaction (HER) and oxygen evolution reaction (OER), represents a promising strategy but remains hindered by sluggish reaction kinetics and limited availability of highly active electrocatalysts especially under alkaline conditions. Addressing this challenge, we successfully synthesized a rGO-VO_2_/W_5_O_14_ (rG-VO_2_/W_5_O_14_) hydrogel electrocatalyst through a facile hydrothermal approach. The prepared composite distinctly reveals an advantageous hierarchical microstructure characterized by VO_2_ nanoflakes uniformly distributed on the surface of rGO nanosheets, intricately integrated with W_5_O_14_ nanorods. Evaluated in a 1.0 M KOH electrolyte, the optimized rG-VO_2_/W_5_O_14_-2 catalyst demonstrates remarkable electrocatalytic performance towards OER, achieving a low overpotential of 265.8 mV and a reduced Tafel slope of 81.9 mV dec^−1^. Furthermore, the catalyst maintains robust stability with minimal performance degradation, exhibiting an overpotential of only 273.0 mV after 5000 cyclic stability tests. The superior catalytic activity and durability are attributed to the synergistic combination of enriched chemical composition, effective electron transfer, and abundant catalytic active sites inherent in the well-optimized rG-VO_2_/W_5_O_14_-2 composite.

## 1. Introduction

Electrochemical water splitting has emerged as a promising and efficient approach for sustainable hydrogen production. This process involves two key half-cell reactions: the hydrogen evolution reaction (HER) and the oxygen evolution reaction (OER) [1,2,3,4,5,6,7,8,9,10,11]. Among these, the OER represents a significant bottleneck due to its complex reaction pathway, which includes a sluggish four-electron transfer mechanism [12]. This inherently slow kinetics results from the need to break O-H bonds and form O=O bonds, thereby necessitating the application of a substantial overpotential to initiate and sustain the reaction efficiently [13]. To overcome these kinetic limitations, extensive research over the past few decades has focused on the rational design and engineering of electrocatalysts. Enhancing catalytic performance has largely depended on tailoring the morphology, crystal structure, and electronic configuration of these materials. Various synthetic strategies have been employed for this purpose, including hydrothermal synthesis, electrodeposition, sol-gel processing, co-precipitation, and anion exchange methods, among others. These techniques aim to optimize surface area, active site exposure, and conductivity, thereby improving the overall efficiency of water splitting systems [14,15]. While noble-metal-based catalysts, such as those incorporating iridium (Ir) and ruthenium (Ru), exhibit exceptional performance in accelerating inherently sluggish catalytic processes still their widespread industrial adoption is significantly constrained by their scarcity, prohibitive costs, and limited availability [16,17,18]. Consequently, considerable research efforts are now dedicated to designing and developing cost-effective, abundant, and efficient alternative materials that can effectively replace these precious metals, thereby facilitating sustainable advancements in renewable energy technologies.

Graphene oxide (GO) is a promising carbon-based nanomaterial characterized by a two dimensional honeycomb lattice consisting primarily of sp^2^-hybridized carbon atoms. Composite materials that integrate graphene with various metals have demonstrated notable energy storage capacities and enhanced cyclic stability. However, the transformation of graphene oxide into reduced graphene oxide (rGO) significantly alters its electrical properties, resulting in electrical conductivity ranging broadly from approximately 2.98 × 10^4^ to 0.1 Sm^−1^ [19]. Among various carbonaceous materials, rGO stands out as particularly advantageous for electrocatalytic applications. Its demand originates from distinctive attributes, including an expanded surface area, superior electron and mass transfer efficiencies, minimized catalyst poisoning, and enhanced charge carrier mobility. The elevated electronic conductivity of rGO is attributed to the efficient transport of delocalized electrons within its conjugated molecular orbitals [19]. Furthermore, the ability of rGO to establish synergistic interactions with other catalytic materials significantly amplifies overall catalytic performance [20,21]. Current research efforts have concentrated extensively on developing non-precious, hierarchical, and heterostructured electrocatalysts with superior catalytic performance, particularly targeting the HER and OER. Prominent candidates include transition-metal-based compounds such as oxides [22,23,24,25,26,27], sulfides [28,29,30,31], nitrides [32,33], and phosphides [34,35]. Among these materials, Vanadium oxides, as a class of transition metal oxides have garnered significant attention in the realm of energy storage and conversion technologies owing to their diverse oxidation states, structural versatility, and rich electrochemical properties. Particularly, the integration of vanadium oxides with carbon-based materials has demonstrated notable synergistic effects resulting in enhanced electrical conductivity, improved structural stability, and superior electrochemical performance. Such vanadium oxide-carbon heterostructures are highly promising candidates for next-generation batteries and supercapacitors, as well as for advanced electrocatalytic applications [36]. Among the various vanadium oxide phases, vanadium dioxide (VO_2_) stands out due to its unique polymorphism and its reversible monoclinic-to-rutile structural phase transition near 67 °C, which imparts intriguing electrical and optical properties [36,37,38,39]. For instance, Usmani et al. synthesized a VO_2_/graphite composite in which VO_2_ was uniformly anchored onto graphite sheets. This heterostructure exhibited an OER overpotential of 470 mV in alkaline electrolyte, indicating considerable catalytic activity [36]. Similarly, Cui et al. reported the fabrication of a Co_3_O_4_/VO_2_ nanohybrid, constructed by anchoring VO_2_ nanoparticles onto Co_3_O_4_ nanocages via a straightforward hydrothermal and subsequent calcination approach. The resulting hybrid demonstrated remarkable electrocatalytic activity, achieving a low OER overpotential of 433 mV at a current density of 10 mA cm^−2^ [40]. In another study, Gowrisankar et al. developed a β-MnO_2_-VO_2_ (M)/2D-rGO composite catalyst, which delivered an overpotential of 334 mV at 10 mA cm^−2^, further highlighting the beneficial role of vanadium oxide-carbon interfaces [41].

**Figure 1:**
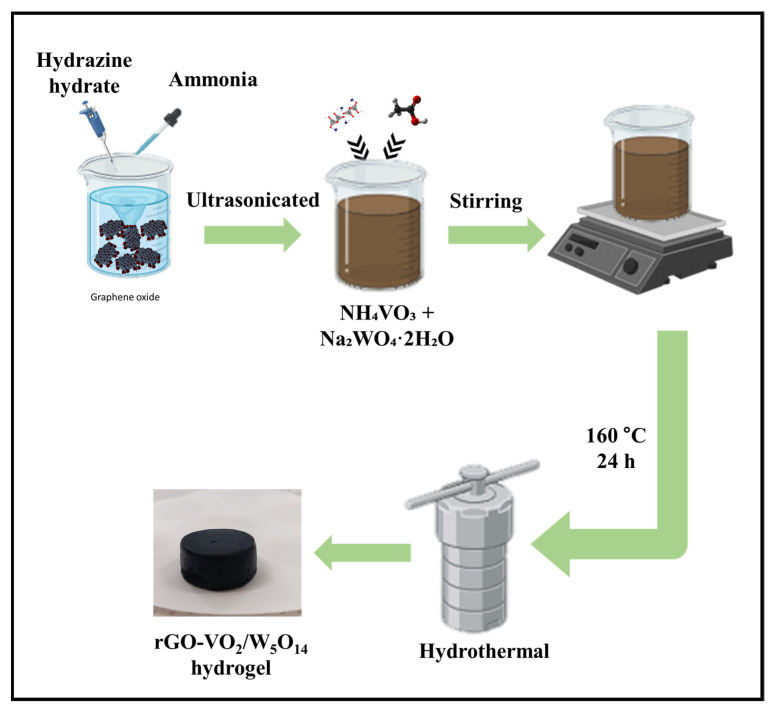
Schematic illustration of synthesis of rGO-VO_2_/W_5_O_14_ hydrogel.

In this study, we present the design and synthesis of rGO-VO_2_/W_5_O_14_ nanostructured electrocatalysts, highlighting their superior electrocatalytic performance toward the OER in alkaline media. The strategic incorporation of W_5_O_14_, derived from rGO-VO_2_ was intended to enhance the intrinsic catalytic activity by promoting favorable structural and electronic interactions within the composite (Figure 1). Comprehensive spectroscopic and microscopic analyses verified the successful formation of the rGO-VO_2_/W_5_O_14_ hybrid, revealing well-dispersed W_5_O_14_ nanorods intimately interconnected with rGO-VO_2_ nanosheets and nanoflakes. Among the series of compositions evaluated, the rG-VO_2_/W_5_O_14_-2 sample exhibited the most outstanding electrochemical performance, achieving a notably low overpotential of 265.8 mV at a current density of 10 mA cm^−2^, accompanied by a Tafel slope of 81.9 mV·dec^−1^. This exceptional activity can be attributed to the enlarged electrochemically active surface area and the pronounced synergistic effects between the constituent phases, which collectively facilitate more efficient charge and mass transfer during OER. Moreover, the robust durability of the rG-VO_2_/W_5_O_14_-2 electrocatalyst was demonstrated through extended cyclic voltammetry and chronopotentiometry tests, confirming its stability under prolonged operational conditions.

## 2. Result and Discussion

**Figure 2:**
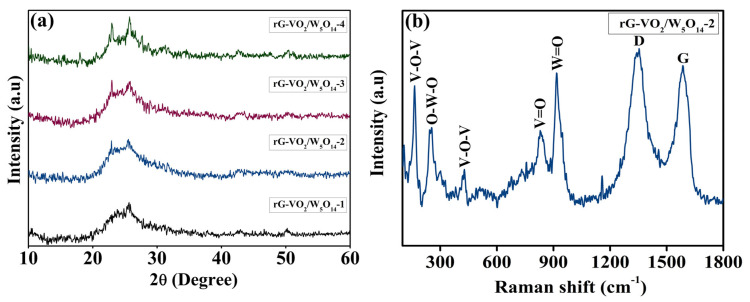
(**a**) XRD spectra of all the composite and (**b**) Raman spectra of rG-VO_2_/W_5_O_14_-2 composite.

The structural integrity, crystallinity, and phase purity of the synthesized materials were thoroughly examined using X-ray diffraction (XRD) analysis. All data were measured at room temperature and recorded in the 2θ range of 10–60°. Figure 2a presents the XRD spectra of rG-VO_2_/W_5_O_14_-1, rG-VO_2_/W_5_O_14_-2, rG-VO_2_/W_5_O_14_-3, and rG-VO_2_/W_5_O_14_-4 composites providing critical insights into their phase composition. The diffraction pattern of rGO exhibits a big hump ranging from 22° to 26° corresponding to the (002) plane and one more peak appeared at 42.5° which shows (111) crystal plane [42]. For the VO_2_, the peaks are assigned at 15.3°, 17.3°, 25.5°, and 31.6°, which correspond to the (200), (−201), (110), and (400) lattice planes. The VO_2_ diffraction peaks provide the confirmation of monoclinic structure which is well matched with the JCPDS No. 00-031-1438 [43]. Additionally, the presence of W_5_O_14_ within the composite is indicated by prominent diffraction peaks observed at 20.5°, 23.3°, 27.4, and 29.6° which are attributed to the (520), (001), (640), and (650) lattice planes. The W_5_O_14_ phase exhibits a tetragonal crystal structure consistent with JCPDS card No. 01-071-0292 [44]. A noticeable trend in the XRD patterns reveals that as the concentration of W_5_O_14_ increases the intensity of the corresponding diffraction peaks also intensifies further validating the successful incorporation of W_5_O_14_ into the composite matrix. These findings confirm the successful formation of the rG-VO_2_/W_5_O_14_ nanocomposite with well-defined crystallinity and phase purity. The structural characteristics as revealed by XRD support the potential applicability of this composite in electrocatalytic processes where well crystallized phases contribute to enhanced catalytic activity and stability. To elucidate the molecular interactions and structural characteristics of the rG-VO_2_/W_5_O_14_-2 nanocomposite, Raman spectroscopy was conducted, as presented in Figure 2b. The Raman spectrum displays, for rGO, two characteristic peaks appear at 1356 and 1586 cm^−1^ corresponding to the D and G bands, respectively [45,46,47]. The D band is indicative of the presence of sp^3^-hybridized carbon atoms and lattice defects, whereas the G band originates from the in-plane vibration of sp^2^-bonded carbon atoms in the graphitic domains. Notably, in the rGO-VO_2_/W_5_O_14_-2 composite, the intensity of the D band surpasses that of the G band suggesting a higher concentration of structural defects and disorder in the rGO lattice. This increased D/G intensity ratio further confirms the partial reduction of GO and the introduction of defect sites which are beneficial for facilitating electron transfer and enhancing the electrocatalytic properties of the composite [46]. In the case for VO_2_, the signal at 164 cm^−1^ corresponds to both V-O-V bending and external wagging modes, while a band observed at 429 cm^−1^ is attributed to the V-O-V stretching mode [37,48]. A peak at 829 cm^−1^ assigned to the V=O stretching vibration of distorted octahedral and distorted square-pyramids, characteristic of VO_2_ [39]. For the W_5_O_14_, the spectrum also exhibits a peak at 254 cm^−1^, representing the bending vibration of O-W-O units and a distinct feature at 916 cm^−1^ which can be ascribed to the terminal W=O bond [49].

**Figure 3:**
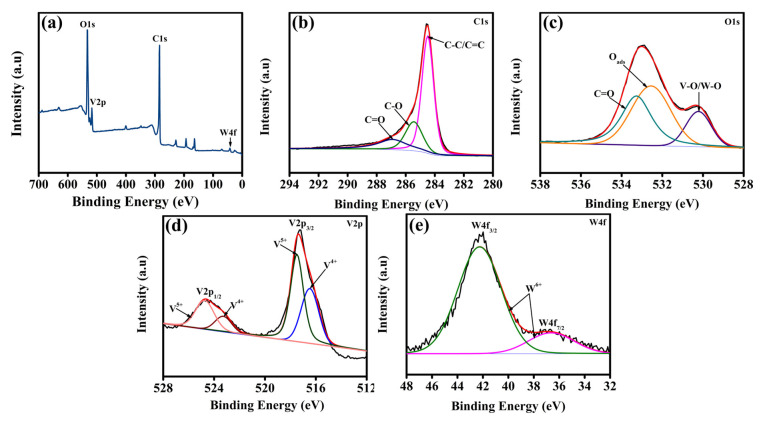
(**a**) XPS survey spectra, high resolution XPS spectra of (**b**) C1s, (**c**) O1s, (**d**) V2p, and (**e**) W4f of rG-VO_2_/W_5_O_14_-2 composite.

X-ray photoelectron spectroscopy (XPS) was employed to meticulously elucidate the chemical states and surface compositions of the synthesized composites. The full XPS survey spectrum (Figure 3a) confirms the presence of carbon (C), oxygen (O), vanadium (V), and tungsten (W) elements within the composite. Figure 3b presents the high-resolution XPS spectrum of C1s, which upon careful deconvolution reveals three distinctive peaks at binding energies of 284.5, 285.6, and 287.5 eV. These peaks are assigned to sp^3^/sp^2^ hybridized C-C/C=C bonds (pink colored line), C-O bonds (green colored line), and (C=O) (Blue colored line), respectively, indicating diverse functional groups present on the rGO surface [50,51]. The high-resolution O1s spectrum depicted in Figure 3c similarly displays multiple oxygen-related species. Precise peak fitting clearly distinguishes characteristic binding energy positions at approximately 530.2 eV (lattice oxygen bonded to V and W atoms, that is V-O/W-O) (pink colored line) (violet colored line), 532.8 eV (surface-adsorbed oxygen species) (orange colored line), and 533.3 eV (C=O functionalities present on the rGO framework), (dark cyan colored line) [43,50,52,53]. Moreover, the detailed V2p core-level spectrum in Figure 3d provides critical insight into the oxidation states of vanadium within the composite structure. Two distinct sets of peaks were identified: one pair centered at 517.4 eV (V2p_3/2_) (green colored line) and 524.6 eV (V2p_1/2_) (green colored line) indicative of V^5+^ oxidation states typical of the VO_2_ lattice, and a second pair located at lower energies, 516.0 eV (V2p_3/2_) and 523.6 eV (V2p_1/2_) attributed to V^4+^ species. This coexistence of multiple vanadium oxidation states implies an enhanced electron transfer capability, essential for effective catalytic activity [41,43]. The W4f high-resolution spectrum (Figure 3e) clearly reveals peaks at binding energies of 36.6 eV (W4f_7/2_) and 42.2 eV (W5p_3/2_). These peaks correspond explicitly to tungsten in its stable W^6+^ oxidation state, confirming successful integration of W atoms into the composite matrix [54,55].

**Figure 4:**
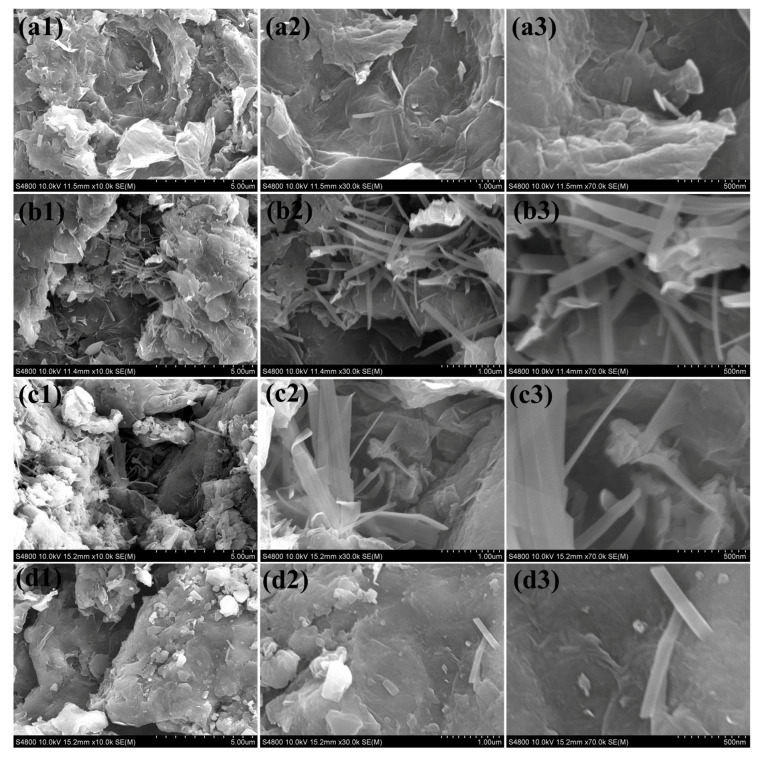
SEM micrograph images of (**a1**–**a3**) rG-VO_2_/W_5_O_14_-1, (**b1**–**b3**) rG-VO_2_/W_5_O_14_-2, (**c1**–**c3**) rG-VO_2_/W_5_O_14_-3, and (**d1**–**d3**) rG-VO_2_/W_5_O_14_-4 electrocatalyst.

The morphology and microstructure of the synthesized rGO-VO_2_/W_5_O_14_ nanocomposites with varying tungsten concentrations were systematically examined using scanning electron microscopy (SEM), as depicted in Figure 4. The SEM images clearly illustrate the notable morphological variations across the different compositions, corresponding to rG-VO_2_/W_5_O_14_-1, rG-VO_2_/W_5_O_14_-2, rG-VO_2_/W_5_O_14_-3, and rG-VO_2_/W_5_O_14_-4. In the rG-VO_2_/W_5_O_14_-1 (Figure 4 (a1–a3)), rGO nanosheets appear as thin and wrinkled structures that act as substrates providing large surface areas and pathways for charge mobility. The nanosheets host small, scattered VO_2_ nanoflakes and sparsely distributed W_5_O_14_ nanorods. Here, the relatively lower concentration of tungsten results in a limited number of W_5_O_14_ nanorods with poor dispersion, thus offering fewer catalytic active sites. The morphology significantly improves at an optimized tungsten concentration in rG-VO_2_/W_5_O_14_-2 as displayed in Figure 4 (b1–b3). This composite distinctly demonstrates a highly favorable hierarchical structure, wherein VO_2_ nanoflakes densely and uniformly decorate the surface of the rGO nanosheets simultaneously interlaced with well-defined, elongated, and abundant W_5_O_14_ nanorods. Such uniform integration of VO_2_ nanoflakes and high-density W_5_O_14_ nanorods on rGO nanosheets substantially enhances the available surface active sites, facilitates efficient electron transfer, and ensures effective electrolyte penetration. These structural attributes collectively contribute to the superior electrocatalytic OER activity observed for this particular composition. Further increase in tungsten content, as depicted in rG-VO_2_/W_5_O_14_-3 (Figure (c1–c3)), leads to excessive growth and partial aggregation of thicker W_5_O_14_ nanorods, overwhelming the VO_2_ nanoflakes and resulting in morphological irregularities. In the rG-VO_2_/W_5_O_14_-4 (Figure (d1–d3)), the microstructure exhibits a noticeable decrease in W_5_O_14_ nanorod density. The fewer, relatively isolated, shorter nanorods along with sparsely distributed VO_2_ nanoflakes on the rGO nanosheets may lead to limited catalytic active site availability, restricted interfacial interactions, and inefficient charge transport, all negatively influencing the electrocatalytic efficiency of this sample.

**Figure 5:**
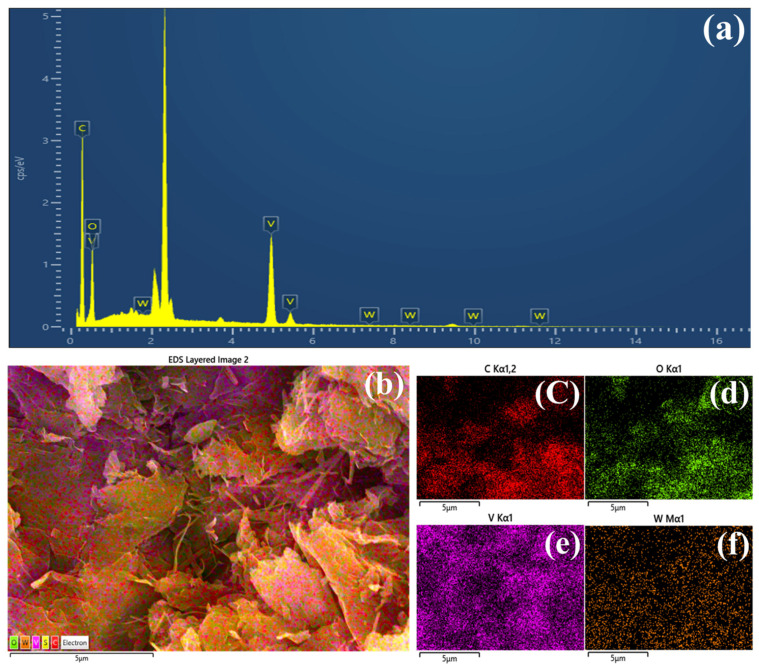
(**a**) Energy-dispersive X-ray spectroscopy analysis of rG-VO_2_/W_5_O_14_-2, and (**b**–**f**) elemental mapping data of rG-VO_2_/W_5_O_14_-2 electrocatalyst.

Elemental composition and distribution within the optimized rG-VO_2_/W_5_O_14_-2 composite were systematically investigated using EDAX analysis, as demonstrated in Figure 5a and Table 1 exhibit the EDAX data of all the samples. The EDAX spectra clearly validated the presence of C, O, V, and W quantitatively establishing their weight percentages as 38.35%, 28.97%, 31.80%, and 0.88%, respectively. To further examine the uniformity and spatial distribution of these constituent elements EDAX elemental mapping was employed, as depicted in Figure 5b–f. The elemental mapping distinctly illustrated a homogeneous dispersion of C, O, V, and W across the entire composite. Such a uniformly distributed elemental configuration promotes efficient interactions between active catalytic sites, potentially facilitating rapid charge transfer pathways and enhanced electrolyte accessibility. Consequently, this homogeneous elemental dispersion significantly contributes to the superior electrocatalytic activity and stability observed in the rG-VO_2_/W_5_O_14_-2 composite during OER processes.

**Figure 6:**
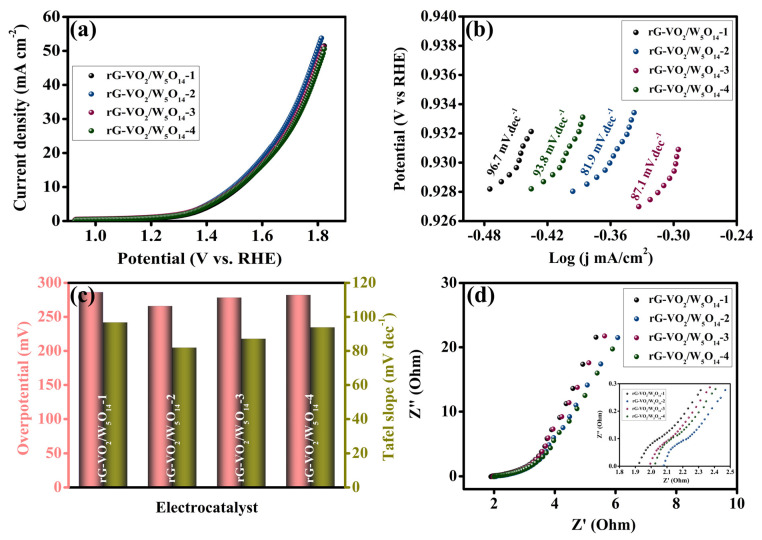
Electrochemical characterizations of electrocatalyst OER performances: (**a**) LSV curves at 5 mV/s scan rate, (**b**) analogous Tafel slopes, (**c**) assessment of the OER performance concerning overpotential at 10 mA cm^−2^ and Tafel slope, and (**d**) EIS spectra of all the electrocatalysts.

To systematically evaluate the oxygen evolution reaction performance of the synthesized electrocatalysts, electrochemical measurements were carried out in a standard three-electrode configuration using 1 M KOH aqueous electrolyte. The LSV was employed to determine the overpotential (η), a key indicator of catalytic activity, with a scan rate of 5 mV s^−1^ and iR compensation applied to all measurements. The LSV profiles of the rG-VO_2_/W_5_O_14_ composites namely rG-VO_2_/W_5_O_14_-1, rG-VO_2_/W_5_O_14_-2, rG-VO_2_/W_5_O_14_-3, and rG-VO_2_/W_5_O_14_-4 are presented in Figure 6a and data presented in Figure 6c. Among them, the rG-VO_2_/W_5_O_14_-2 sample exhibited the most remarkable OER activity, achieving a low overpotential of 265.8 mV at a benchmark current density of 10 mA cm^−2^. In contrast, higher overpotentials were observed for rG-VO_2_/W_5_O_14_-1 (286.3 mV), rG-VO_2_/W_5_O_14_-3 (278.3 mV), and rG-VO_2_/W_5_O_14_-4 (282.1 mV) indicating inferior electrocatalytic performance under identical conditions. The superior performance of rG-VO_2_/W_5_O_14_-2 can be attributed to the optimized composition and synergistic integration of W_5_O_14_ which enhances the density of active sites and facilitates faster electron transfer. This trend suggests a direct correlation between W_5_O_14_ concentration in the hybrid material and OER efficiency. To further elucidate the reaction kinetics, Tafel slope analysis was conducted, as shown in Figure 6b,c. The rG-VO_2_/W_5_O_14_-2 catalyst recorded the lowest Tafel slope of 81.9 mV dec^−1^, reflecting more favorable charge transfer kinetics. This value was lower than those obtained for rG-VO_2_/W_5_O_14_-1 (96.7 mV dec^−1^), rG-VO_2_/W_5_O_14_-3 (87.1 mV dec^−1^), and rG-VO_2_/W_5_O_14_-4 (93.8 mV dec^−1^), confirming the enhanced intrinsic electrocatalytic activity of rG-VO_2_/W_5_O_14_-2. Electrochemical impedance spectroscopy (EIS) was employed to investigate the charge transfer properties and interfacial resistance of the catalysts during OER. The Nyquist plots displayed in Figure 6d reveal that rG-VO_2_/W_5_O_14_-2 exhibits the smallest semicircle diameter, signifying reduced charge transfer resistance (R_ct_) and accelerated interfacial electron transport. This behavior is further supported by the inset of Figure 6d, where the real impedance (Zre) trends toward lower values. The extracted R_ct_ values for rG-VO_2_/W_5_O_14_-1, rG-VO_2_/W_5_O_14_-2, rG-VO_2_/W_5_O_14_-3, and rG-VO_2_/W_5_O_14_-4 were 0.16, 0.12, 0.13, and 0.14 Ω, respectively. The notable decrease in R_ct_ for rG-VO_2_/W_5_O_14_-2 underscores its superior electrochemical performance, which is primarily attributed to enhanced conductivity and the presence of abundant surface-active sites facilitated by the rGO-VO_2_/W_5_O_14_ integration. This synergistic effect promotes efficient electron transport pathways and increases the rate of oxygen evolution, making rG-VO_2_/W_5_O_14_-2 a promising electrocatalyst for OER applications.

**Figure 7:**
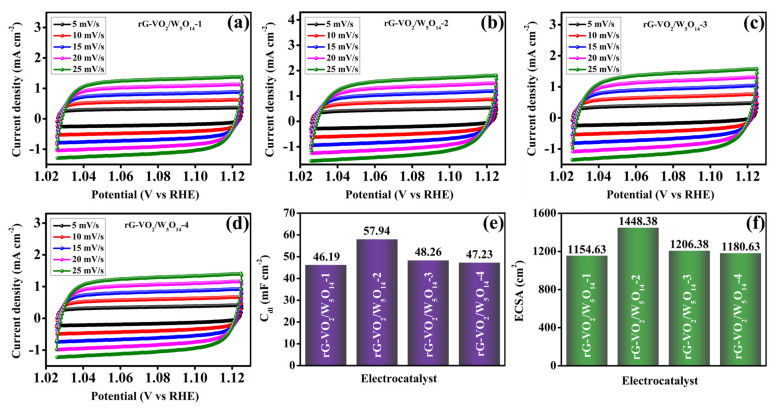
Cyclic voltammetry analysis at different scan rate (**a**) rG-VO_2_/W_5_O_14_-1, (**b**) rG-VO_2_/W_5_O_14_-2, (**c**) rG-VO_2_/W_5_O_14_-3, and (**d**) rG-VO_2_/W_5_O_14_-4, (**e**) C_dl_ graph, and (**f**) ECSA graph of all the electrocatalysts.

Figure 7 illustrates the cyclic voltammetry (CV) profiles of the rG-VO_2_/W_5_O_14_ composites with varying W_5_O_14_ ratios, recorded at different scan rates. The CV curves for rG-VO_2_/W_5_O_14_-1 (Figure 7a), rG-VO_2_/W_5_O_14_-2 (Figure 7b), rG-VO_2_/W_5_O_14_-3 (Figure 7c), and rG-VO_2_/W_5_O_14_-4 (Figure 7d) reveal a progressive increase in current response with increasing scan rate. This trend underscores the dependence of electrocatalytic behavior on scan rate, suggesting improved charge transport kinetics across all samples. To quantify the electrochemically active surface area (ECSA), the double-layer capacitance (C_dl_) was evaluated from the CV curves at varying scan rates, as shown in Figure 7e. The calculated C_dl_ values for rG-VO_2_/W_5_O_14_-1, rG-VO_2_/W_5_O_14_-2, rG-VO_2_/W_5_O_14_-3, and rG-VO_2_/W_5_O_14_-4 were found to be 46.19, 57.94, 48.26, and 47.23 mF cm^−2^, respectively. These values directly reflect the extent of surface area available for electrochemical reactions. Subsequently, the ECSA values were estimated using the relation ECSA = C_dl_/C_s_, where C_s_ represents the specific capacitance of a flat surface (typically ~0.040 mF cm^−2^ for KOH) [56]. As depicted in Figure 7f, the corresponding ECSA values for rG-VO_2_/W_5_O_14_-1, rG-VO_2_/W_5_O_14_-2, rG-VO_2_/W_5_O_14_-3, and rG-VO_2_/W_5_O_14_-4 were calculated to be 1154.63, 1448.38, 1206.38, and 1180.63 cm^2^, respectively. Among the samples, rG-VO_2_/W_5_O_14_-2 exhibited the highest ECSA indicating a greater number of electrochemically accessible active sites. This enhancement is attributed to the optimal incorporation of W_5_O_14_ within the composite matrix, which promotes a more effective interface between the rGO, VO_2_, and W_5_O_14_ nanostructures. The expanded active surface area plays a critical role in facilitating charge transfer and catalytic efficiency, thereby contributing to the improved overall electrochemical performance of the OER.

**Figure 8:**
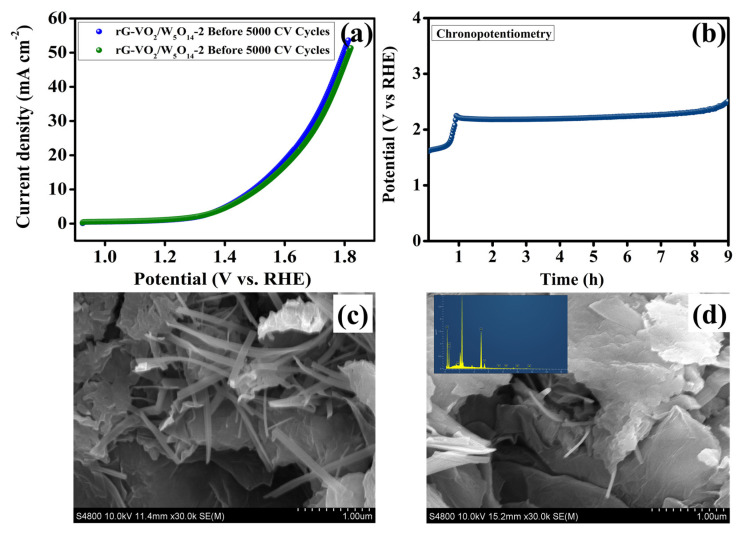
(**a**) LSV curves of rGO-VO_2_/W_5_O_14_-2 before and after 5000 CV cycles, (**b**) Chronopotentiometry analysis, SEM images (**c**) Before analysis, and (**d**) After analysis (inset EDAX analysis).

The long term stability of electrocatalysts is a crucial factor in electrochemical performance evaluation, particularly for potential commercial applications. To assess the structural and electrochemical durability of the heterostructured rG-VO_2_/W_5_O_14_-2 electrocatalyst in a 1.0 M KOH electrolyte, CV studies and chronopotentiometry measurements were conducted. The CV cycling test was performed at a scan rate of 50 mV s^−1^ in a 1.0 M KOH electrolyte to monitor the durability of the electrocatalyst under prolonged operational conditions. As depicted in Figure 8a, the polarization curve of rG-VO_2_/W_5_O_14_-2 was recorded before and after 5000 CV cycles to analyze its performance retention. After undergoing 5000 cycles, the electrocatalyst maintained an overpotential of 273.0 mV at a current density of 10 mA cm^−2^ which closely matches the initial values obtained before cycling, demonstrating excellent electrochemical stability. Furthermore, a chronopotentiometry experiment was conducted at a current density of 10 mA cm^−2^ over a continuous for 9 h (Figure 8b) to further examine the catalyst’s endurance. At the start of the chronopotentiometry test, the electrode surface conditions itself, its outer layer becomes more oxidized and oxygen bubbles temporarily cover parts of the surface. Both effects briefly make the reaction harder, so the cell voltage rises. Once the surface and wetting stabilize and bubbles detach regularly, the potential levels off and stays steady. The rG-VO_2_/W_5_O_14_-2 electrocatalyst exhibited remarkable stability. However, a slight loss in performance could be attributed to several factors, including the minor leaching of active materials from the electrode’s surface, or potential structural degradation due to oxidation under high potentials [57,58]. Figure 8c,d shows the before and after analysis SEM images. The SEM micrographs reveal that the hierarchical nanostructure VO_2_ nanoflakes anchored on rGO and intertwined with W_5_O_14_ nanorods remains intact after, indicating negligible structural degradation. Consistently, EDX (inset) of the C, O, V, and W exhibiting weight percentage of 29.02%, 25.94%, 44.27%, and 0.76 shows a vanadium increasing from that is no loss of V from the surface; thus, oxidation to V^5+^ manifests as a benign surface reconstruction rather than vanadate leaching.

## 3. Conclusions

In this study, we have successfully synthesized a novel rGO-VO_2_/W_5_O_14_ electrocatalyst via a facile hydrothermal method, showcasing remarkable potential for OER applications. The synthesized rGO-VO_2_/W_5_O_14_ composite demonstrates a homogeneous and uniform integration of its constituents which significantly contributes to its enhanced electrocatalytic activity. Among the prepared samples, the rG-VO_2_/W_5_O_14_-2 composition exhibited exceptional catalytic performance requiring a notably low overpotential of 265.8 mV to achieve a current density of 10 mA cm^−2^. This superior electrocatalytic performance is primarily attributed to the synergistic effects arising from the optimized structural interactions among rGO nanosheets, VO_2_ nanoflakes, and W_5_O_14_ nanorods, which effectively facilitate electron transport and amplify catalytic activity. Furthermore, all synthesized rGO-VO_2_/W_5_O_14_ composites displayed significantly reduced charge transfer resistance, promoting rapid electron and OH^−^ ion movement across the electrode interface, thereby accelerating OER kinetics. This work presents an efficient approach to developing advanced electrocatalysts with superior charge transfer dynamics and elevated electrochemical performance. Consequently, the rGO-VO_2_/W_5_O_14_ hydrogel-based materials exhibit considerable potential for practical utilization in advanced energy conversion and storage technologies.

## 4. Experimental Section

### 4.1. Chemicals

Sodium tungstate dihydrate (Na_2_WO_4_·2H_2_O, ≥99%), ammonium metavanadate (NH_4_VO_3_, ≥99%), polyvinylidene fluoride (PVDF), and N-methyl-2-pyrrolidone (NMP, ≥99%) were procured from Sigma-Aldrich St. Louis, MO, USA. Hydrochloric acid (HCl, extra pure), ammonia (NH_3_, extra pure), and ethanol (EtOH, C_2_H_5_OH, 94.5%) were sourced from Duksan Chemicals, Gyeonggi-do, Republic of Korea. Potassium hydroxide (KOH, >85%) was supplied by DaeJung Chemicals & Metals, Gyeonggi-do Republic of Korea. Acetylene black (99.9+%) was obtained from Thermo Scientific, Seoul, Republic of Korea. The carbon cloth utilized in this study was acquired from NARA Cell-Tech Corporation, Seoul, Republic of Korea. All chemicals were used without further purification, and deionized (DI) water was exclusively employed in all experimental procedures to ensure consistency and reliability in the study.

### 4.2. Preparation of rGO-VO_2_/W_5_O_14_ Composite

The rGO-VO_2_/W_5_O_14_ electrocatalyst was synthesized via a facile one-step hydrothermal approach, as schematically illustrated in Figure 1. Initially, 100 mg of graphene oxide (GO) was dispersed in 20 mL of DI water through ultrasonication to achieve a homogeneous suspension. Subsequently, 4.5 mL of ammonia solution and 150 μL of hydrazine hydrate were introduced into the mixture under continuous stirring to facilitate reduction and pH adjustment.

Following this, 0.043 g of NH_4_VO_3_ was added to the GO dispersion, and the mixture was further ultrasonicated for 10 min to ensure thorough mixing and precursor dissolution. To this solution, four distinct concentrations of Na_2_WO_4_·2H_2_O specifically 0.088, 0.176, 0.3525, and 0.52887 g were successively introduced. The total volume of the reaction mixture was then adjusted to 30 mL by the addition of 10 mL DI water. The resulting suspension underwent a further 10 min of ultrasonication, followed by magnetic stirring for 1 h to promote precursor interaction. The fully prepared solution was subsequently transferred into a Teflon-lined stainless-steel autoclave and subjected to hydrothermal treatment at 160 °C for 24 h, enabling the formation of a self-supporting hydrogel. After the hydrothermal process, the resulting hydrogel was subjected to freeze-dried for 24 h to obtain the final composite. For the preparation of rGO-VO_2_/W_5_O_14_ composites, the aforementioned procedure was repeated with the same four concentrations of Na_2_WO_4_·2H_2_O. The resulting samples were denoted as rG-VO_2_/W_5_O_14_-1, rG-VO_2_/W_5_O_14_-2, rG-VO_2_/W_5_O_14_-3, and rG-VO_2_/W_5_O_14_-4, corresponding to increasing sodium tungstate precursor amounts.

### 4.3. Material Characterization

The structural properties of the synthesized nanomaterials were examined using an X-ray diffractometer (X’Pert Pro, PAN Analytical, Almelo, The Netherlands) equipped with a Cu Kα radiation source. Raman spectroscopy was carried out with an XploRA Plus system (HORIBA Jobin Yvon S.A.S, Paris, France) to analyze the vibrational characteristics. The surface chemical composition and oxidation states were investigated through X-ray photoelectron spectroscopy (XPS) using a Thermo Scientific K-Alpha surface analysis system. Additionally, the surface morphology, particle size distribution, elemental composition, and elemental mapping were evaluated using scanning electron microscopy (SEM, HITACHI S-4800, Tokyo, Japan) integrated with an energy-dispersive X-ray (EDX) analysis system.

### 4.4. Electrochemical Analysis

Electrochemical measurements were systematically conducted using a Biologic Instrument WBCS3000 battery cycler (Gières, France) to comprehensively evaluate electrocatalytic performance. A conventional three-electrode configuration was adopted, consisting of carbon cloth (CC) as the working electrode, a Hg/HgO reference electrode, and a platinum plate as the counter electrode. Prior to catalyst deposition, the carbon cloth substrates underwent rigorous pretreatment involving successive sonication steps in 1 M HCl, deionized water, and ethanol, each for 20 min, followed by overnight drying at 70 °C to ensure cleanliness and optimal surface properties.

The electrocatalyst slurry was meticulously prepared using an optimized weight ratio of 80% active material, 10% polyvinylidene fluoride (PVDF), and 10% acetylene black dispersed uniformly in N-methyl-2-pyrrolidone (NMP) solvent. This slurry was evenly coated onto the pretreated carbon cloth substrates (1 × 1 cm^2^) and subsequently dried overnight at 60 °C to ensure robust adhesion and homogeneity.

Electrochemical characterization was executed in a nitrogen-saturated 1 M KOH aqueous electrolyte. Cyclic voltammetry (CV) measurements were performed within a non-Faradaic potential window of 0.1 to 0.2 V at various scan rates (5, 10, 15, 20, and 25 mV s^−1^) to investigate the electrochemical active surface area (ECSA) and capacitive behavior. Linear sweep voltammetry (LSV) analysis, conducted at a scan rate of 5 mV s^−1^ across a potential range of 0 to 1 V, was employed to determine the overpotential required for the OER. The measured potentials versus Hg/HgO reference electrode were accurately converted to the reversible hydrogen electrode (RHE) scale using the following Nernst equation:

E_RHE_ = E_Hg/HgO_ + E°_Hg/HgO_ + 0.0591 × (pH) (1)

where E°_Hg/HgO_ represents the standard electrode potential of the Hg/HgO reference electrode, and the pH of a 1 M KOH solution is approximately 13.9. LSV was utilized to relate the optimized electrocatalysts stability before and after 5000 cycles of CV study.

## Figures and Tables

**Table 1:** EDAX analysis data of all the electrocatalysts.

Electrocatalysts	C (Wt%)	O (Wt%)	V (Wt%)	W (Wt%)
rG-VO_2_/W_5_O_14_-1	39.77	29.7	30.13	0.40
rG-VO_2_/W_5_O_14_-2	38.35	28.97	31.80	0.88
rG-VO_2_/W_5_O_14_-3	37.78	29.91	30.76	1.55
rG-VO_2_/W_5_O_14_-4	38.15	30.79	28.26	2.8

## Data Availability

The data presented in this study are available on request from the corresponding author.
